# Fewer Basins Will Follow Their Budyko Curves Under Global Warming and Fossil‐Fueled Development

**DOI:** 10.1029/2021WR031825

**Published:** 2022-08-22

**Authors:** Fernando Jaramillo, Luigi Piemontese, Wouter R. Berghuijs, Lan Wang‐Erlandsson, Peter Greve, Zhenqian Wang

**Affiliations:** ^1^ Department of Physical Geography and Bolin Centre for Climate Research Stockholm University Stockholm Sweden; ^2^ Baltic Sea Centre Stockholm University Stockholm Sweden; ^3^ Department of Agricultural, Environmental, Food and Forestry Science and Technology University of Florence Florence Italy; ^4^ Department of Earth Sciences Free University Amsterdam Amsterdam The Netherlands; ^5^ Stockholm Resilience Centre Stockholm University Stockholm Sweden; ^6^ International Institute for Applied Systems Analysis Water Program Laxenburg Austria; ^7^ Key Laboratory of Western China's Environmental Systems (Ministry of Education) College of Earth and Environmental Sciences Lanzhou University Lanzhou China

**Keywords:** Budyko, global warming, evaporative ratio, aridity index, CMIP6

## Abstract

The Budyko framework consists of a curvilinear relationship between the evaporative ratio (i.e., actual evaporation over precipitation) and the aridity index (i.e., potential evaporation over precipitation) and defines evaporation's water and energy limits. A basin's movement within the Budyko space illustrates its hydroclimatic change and helps identify the main drivers of change. On the one hand, long‐term aridity changes drive evaporative ratio changes, moving basins along their Budyko curves. On the other hand, historical human development can cause river basins to deviate from their curves. The question is if basins will deviate or follow their Budyko curves under the future effects of global warming and related human developments. To answer this, we quantify the movement in the Budyko space of 405 river basins from 1901–1950 to 2051–2100 based on the outputs of seven models from the Coupled Model Intercomparison Project ‐ Phase 6 (CMIP6). We account for the implications of using different potential evaporation models and study low‐ and high‐emissions scenarios. We find considerable differences of movement in Budyko space regarding direction and intensity when using the two estimates of potential evaporation. However, regardless of the potential evaporation estimate and the scenario used, most river basins will not follow their reference Budyko curves (>72%). Furthermore, the number of basins not following their curves increases under high greenhouse gas emissions and fossil‐fueled development SP585 and across dry and wet basin groups. We elaborate on the possible explanations for a large number of basins not following their Budyko curves.

## Key Points

- We quantify CMIP6‐simulated movements in Budyko space of 405 river basins from 1901 to 2100
- Most river basins will not follow their original Budyko‐curve trajectories
- The number of basins that do not follow their Budyko trajectories increases with the effects of global warming

## 1 Introduction

Assessing future shifts in water resources and securing these resources through adaptation and mitigation requires an understanding of hydroclimatic change (Baldassarre et al., 2015; Brown et al., 2019; Nissan et al., 2019; Sivapalan & Blöschl, 2015). For decades, the Budyko framework (Budyko, 1948, 1974) has been used to understand hydroclimatic change by studying the relationship between water and energy available on the land surface and considering evaporation's water and energy limits. The framework provides a curvilinear relationship between the long‐term means of the evaporative ratio (i.e., actual evaporation over precipitation) and the aridity index (i.e., potential evaporation over precipitation). Many Budyko studies have focused on understanding the physical and hydrological mechanisms underlying basins' locations in the Budyko space (e.g., Berghuijs et al., 2014, 2020; Gan et al., 2021; C. Wang et al., 2016; Xu et al., 2013) or developing stochastic and deterministic approaches that quantify the sensitivity of water resources to climatic conditions (Berghuijs et al., 2017; Z. Chen et al., 2021; Gudmundsson et al., 2016; Liu et al., 2019; Roderick & Farquhar, 2011).

**Figure 1:**
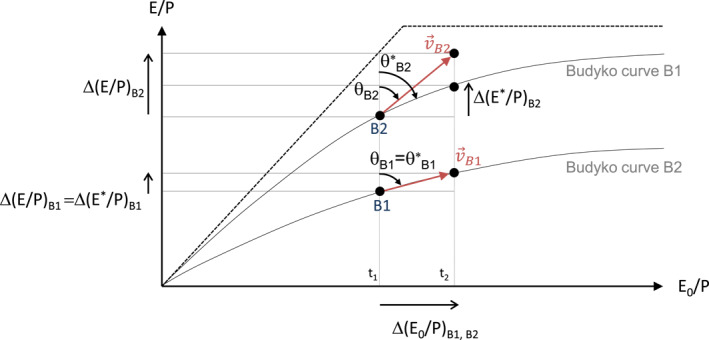
Following or deviating from the Budyko‐curve trajectory between periods 1 (t1) and 2 (t2). For the first case, hydrological basin Nr. 1 (B1) moves along the Budyko curve with a vector of movement ${\vec{v}}_{B1}$ with change in the aridity index as its horizontal component Δ(E_0_/P)_B1_ and change in the evaporative ratio, Δ(E/P)_B1_ as its vertical component. The magnitude of the vector $\left|{\vec{v}}_{B1}\right|$ gives the intensity (I_B1_) of the movement (scalar), and the inclination angle clockwise from the vertical (θ_Β1_) gives the direction of movement. For the second case, the hydrological basin Nr. 2 (B2) deviates from the trajectory of its original Budyko with a vector of movement ${\vec{v}}_{B2}$, with change in the aridity index (E_0_/P)_B2_ as its horizontal component and change in the evaporative ratio Δ(E/P)_B2_ as its vertical component, with direction of movement θ_B2_ and intensity I_B2_. In this example, the evaporative ratio change Δ(E_0_/P)_B2_ is larger than expected when accounting for changes in the aridity index only (i.e., following the Budyko curve of B1; Δ(E*/P)_B1_), which would otherwise result in the direction of movement (θ*_B2_) and a magnitude (I*_B2_).

Although Budyko's initial relationship is spatial (i.e., between river basins), many scientific developments have used it to quantify and attribute temporal hydroclimatic changes to particular drivers. The studies mostly use the concepts of elasticity (Němec & Schaake, 1982; Sankarasubramanian et al., 2001; Schaake, 1990), decomposition (D. Wang & Hejazi, 2011), separation (Destouni et al., 2013) and sensitivity (Dooge, 1992) to differentiate and quantify the drivers of runoff or evapotranspiration changes. Almost all techniques express the change in the variable of interest per change of driving climatic conditions such as temperature, rainfall, and potential evaporation. These concepts assume that every hydrological basin on Earth has a set of combinations of evaporative ratio and aridity index related to its characteristics of vegetation, soils, topography, climate seasonality, and snow‐to‐rain ratio, which make up its Budyko curve (Figure 1) (L. Zhang et al., 2001). Hence, changing any of these characteristics may move the hydrological basin into another Budyko curve.

The mathematical space spanned by the evaporative ratio and the aridity index is often named the Budyko space (e.g., Greve et al., 2015; Gudmundsson et al., 2016; Jaramillo & Destouni, 2014; Moussa & Lhomme, 2016) and the change in the location in the Budyko space of a particular region or basin can be referred to as “movement in Budyko space” (Figure 1). Both concepts help visualize temporal hydroclimatic change in a given hydrological basin. The movement corresponds to the joint change in the aridity index and evaporative ratio between two time periods for a specific hydrological basin (Destouni et al., 2013; Jaramillo & Destouni, 2014; Jaramillo et al., 2018; van der Velde et al., 2013), with the limits of energy (aridity index = evaporative ratio) and water availability (evaporative ratio = 1) defining the convex and asymptotical shape of the Budyko curves and the type of movements in the Budyko space (Greve et al., 2015). A hydrological basin under stable and uniform landcover and water storage conditions is expected to move along its Budyko‐type curve if a change in the aridity index is the main driver of evaporative‐ratio changes between the two periods (Tang & Wang, 2017).

However, as water use and storage have historically altered the evaporative ratio of many basins worldwide (Jaramillo & Destouni, 2014; D. Wang & Hejazi, 2011), the temporal movements of river basins are not necessarily restricted to the Budyko‐curve trajectories. For instance, under stable conditions of the aridity index, land conversion from forest to grassland decreases evaporation and root zone capacity, increasing runoff and decreasing the evaporative ratio (Nijzink et al., 2016; Sterling et al., 2013). In other words, a basin experiencing such wide‐scale land conversion would move vertically downwards in the Budyko space without any other driver. On the contrary, a basin under conversion from grassland to forest cover would increase evapotranspiration and typically move upwards in the Budyko space (Donohue et al., 2007). Other examples of upward movements in Budyko space include the expansion of irrigation (Jaramillo & Destouni, 2015; D. Wang & Hejazi, 2011), the impounding effects of reservoirs on rivers (Levi et al., 2015), forest management (Jaramillo et al., 2018), or changes from snow to rain which decrease runoff and translate into an upward movement in the Budyko space (Berghuijs et al., 2014).

Moreover, global warming may also alter the movement of a river basin in Budyko space. Increased concentrations of greenhouse gases decrease the outgoing longwave radiation and increase absorbed solar radiation, accumulating energy in the climate system and warming the planet (Donohoe et al., 2014). Furthermore, the increase in temperatures leads to a rise in vapor pressure deficit, which in combination with the Clausius–Clayperon relationship (i.e., a nonlinear increase of saturation vapor pressure as a function of temperature), is expected to increase aridity. The question is whether global warming will make river basins follow their original Budyko curves or deviate from them, as observed in historical studies (Jaramillo & Destouni, 2014). To answer this question, we calculate movements in Budyko space between 1901‐1950 and 2051–2100 for 405 large river basins worldwide under high and low greenhouse gas emission scenarios. We use the outputs of seven models of the Coupled Model Intercomparison Project ‐ Phase 6 (CMIP6), and acknowledging that the parametrization of potential evaporation may heavily influence such movement, we use two different estimates for its quantification.

## 2 Methods

### 2.1 Data

We selected seven widely‐used Earth System Models (ESMs) of the CMIP6 with sufficient data availability from 1901 to 2100 to allow the computation of potential evaporation (E_0_), actual evaporation (E), and precipitation (P) (Table 1). The ESMs have different spatial resolutions and, in some cases, integrate different land surface and dynamic vegetation models. Moreover, we included a coupled model, the EC‐Earth3‐Veg, as it has two configurations with and without dynamic vegetation, which helps study any implication of changing vegetation for the movement of basins in Budyko space. The variables describe the aridity index (E_0_/P) and the evaporative ratio (E/P). We calculated the mean areal values of E_0_, P, and E for the largest 405 river basins available in the Global Runoff Database Center GRDC (grdc@bafg.de); being the Skjern Å in Denmark the smallest hydrological basin (∼3,000 km^2^) and the largest the Amazon (∼5.9 million km^2^). It is worth noting that some hydrological basins are smaller than the actual resolution of most ESMs; for instance, 19 hydrological basins are smaller than 8,000 km^2^; the spatial resolution of the ESM with the highest resolution (Table 1). The CMIP6 monthly data was downloaded from the Earth System Grid (ESG, https://esgf-node.llnl.gov/search/cmip6/), using various realizations for accounting for all CMIP6 outputs needed in the analysis. The monthly data was used to compute annual means for the historical 50‐year period 1901–1950 and the far future 2051–2100. Long‐term and separated periods are usually used to determine the implications of climate change, such as in the IPCC reports (e.g., IPCC, 2014) and other studies (e.g., Gudmundsson et al., 2021; Jaramillo & Destouni, 2014; Milly et al., 2005). While the historical experiment of the CMIP6 fully covers the first period, the second is fully covered by the Shared Socioeconomic Pathways (SSPs) experiments. We focused on the low‐emission SSP126 and high‐emission SSP585 CMIP6 experiments as their comparison insights into the hydroclimatic impacts of global warming. The first corresponds to an additional radiative forcing of 2.6 W/m^2^ by 2100, taking the green road with small challenges to mitigation and adaptation (lowest emission rate scenario), and the second to 8.5 W/m^2^ by 2100 without carbon emission mitigation strategies (highest emission rate scenario).

**Table 1:** The Seven CMIP6 Models Used in the Study

Model	Institute	Dynamic vegetation	Land surface model	Ensemble member	Horizontal resolution (lon × lat)	Reference
ACCESS‐ESM1‐5	Australian Community Climate and Earth System Simulator	CABLE	CABLE	r1i1p1f1	1.88° × 1.24°	Ziehn et al., 2020
CESM2	National Center for Atmospheric Research	None	CLM5.0	r4i1p1f1	1.25° × 0.94°	Danabasoglu et al., 2020
EC‐Earth3	European Research Consortium	None	HTESSEL	r1i1p1f1	0.70° × 0.70°	Döscher et al., 2022
EC‐Earth3‐Veg	European Research Consortium	LPJ‐GUESS	HTESSEL	r1i1p1f1	0.70° × 0.70°	Döscher et al., 2022
HadGEM3‐GC31‐MM	Met Office Hadley Center	None	Global Land 7.0	r1i1p1f3	0.83° × 0.56°	Walters et al., 2019
IPSL‐CM6A‐LR	Institution Pierre‐Simon Laplace	ORCHIDEE	ORCHIDEE	r1i1p1f1	2.50° × 1.26°	Boucher et al., 2020; Cheruy et al., 2020
MPI‐ESM1‐2‐HR	Max Planck Institute for Meteorology	None	JSBACH	r1i1p1f1	0.94° × 0.94°	Mauritsen et al., 2019; Müller et al., 2018

#### 2.1.1 Aridity Index

Precipitation data was obtained from the CMIP6 output for precipitation flux (CMIP6 variable name: pr). Since the value of the aridity index is heavily dependent on the E_0_ model applied for its calculation (Greve et al., 2019), we use two different methods known to differ from each other for its estimation; the first being the energy‐only method (E_0‐EO_; Milly & Dunne [2016]) and the second the Penman‐Monteith for open water (E_0‐PM_; Maidment [1993]). The E_0‐EO_ is the original method used by Budyko within his framework (Budyko, 1948, 1974) and outperforms other methods when using climate model outputs (Equation 1). The energy‐only method uses net radiation as the energy constraint to evaporation and is expressed as

(1) $$E_{0-EO}=0.8\left(R_{n}-G\right)$$

The R_n_ is net radiation divided by the latent heat of vaporization, and G is the ground heat flux into the subsurface, both in mm/day. Since G is not available as a CMIP6 output, we estimated the term Rn – G in two ways. First, as the difference between CMIP6 outputs for downwelling short (*rsds*) and long (*rlds*), and upwelling short (*rsus*) and long (*rlus*) wave radiation (*rsds* + *rlds* ‐ *rsus* – *rlus*), assuming G to be zero as it is not provided by the CMIP6 models (Cook et al., 2014). Second, we estimated Rn – G as the sum of the CMIP6 outputs of latent (*hfls)* and sensible (*hfss*) heat fluxes (Milly & Dunne, 2016). The constant 0.8 in Equation 1 reflects the fraction of available energy (∼80%) going into latent heat flux (Koster & Mahanama, 2012), and its derivation has been described by Yang & Roderick (2019).

The second potential evaporation model, E_0‐PM,_ calculates E_0_ as

(2) $$E_{0-PM}=\frac{\Delta }{\Delta +\gamma }\left(R_{n}-G\right)+\frac{\gamma }{\Delta +\gamma }\frac{6.43\left(1+0.536u\right)\left(e_{s}-e_{a}\right)}{L_{v}}$$

where Rn and G are in mm/day, u is the wind speed at 2 m in ms^−1^, e_s_ − e_a_ is the saturation vapor pressure deficit in kPa, calculated as

(3) $$e_{s}=0.6108e^{17.27\;T/\left(T+237.3\right)}$$

(4) $$e_{a}=\frac{R_{h}e_{s}}{100}$$

with T as the air temperature at 2 m height in deg C and R_h_ is mean relative humidity, and Pre is surface atmospheric pressure in kPa. In turn, γ is the psychrometric constant in kPadegC^−1^ calculated as

(5) $$\gamma =\frac{0.0016286Pre}{L_{v}}$$

where Pre is surface atmospheric pressure in kPa and the L_v_ the latent heat of vaporization of water in MJkg^−1^ calculated as

(6) $$L_{v}=2.501-0.002361T$$

Finally, the slope of the saturation vapor pressure curve with respect to temperature Δ in kPadegC^−1^ is calculated as

(7) $$\Delta =\frac{4098e_{s}}{{\left(T+237.3\right)}^{2}}$$

The CMIP6 outputs used were *tas* for T, *sfcWind* for u_2_, *ps* for Pre, and *hurs* for R_h_.

The 50‐year E_0‐EO_ and E_0‐PM_ annual means for 1901–1950 and 2051–2100 were divided by the corresponding 50‐year annual mean of P to obtain the 50‐year annual mean of the aridity index (E_0_/P). The difference in E_0_/P between the two periods, Δ(E_0_/P), is the horizontal component of the movement in Budyko space.

#### 2.1.2 Evaporative Ratio

We estimated actual evaporation for the 50‐year periods in two ways. The first was directly taken from the monthly CMIP6 output of evaporation (E), which includes sublimation and transpiration (*evspspl*). We aggregated this output annually to calculate the 50‐year annual estimate for the periods 1901–1950 and 2051–2100. We removed coastal grid cells in each hydrological basin to remove the effect of ocean evaporation on *evspspl*.

The second way was the actual evaporation estimate (E*), referred to here as the climate estimate since it is estimated in terms of the aridity index via the “Budyko‐type” model of Yang et al. (2008).

(8) E∗=P1+E0P−n−1/n

Where *n is* the parameter that best represents the characteristics of the hydrological basin during the 50 years (e.g., vegetation, soils, topography, seasonality in precipitation and potential evaporation, snow‐rain characteristics) and E_0_/P is the aridity index (L. Zhang et al., 2001). This index was calculated for each period as the ratio of the 50‐year mean annual potential evaporation (E_0‐EO_ or E_0‐PM_) by the 50‐year mean annual precipitation mean. We estimated a mean n‐value representing the original conditions of each basin by using the 50‐year mean annual data of E, E_0_ and P for the initial period 1901–1950 in Equation 8. For the E* value of the future period 2051–2100, we used the 2051–2100 mean of E_0_ divided by the 2051–2100 mean of P, but with the previously calibrated n‐value (1901–1950).

The difference in the resulting evaporative ratios E* of the two periods gives the evaporative ratio change Δ(E*/P); the vertical component of the movement of change expected only from aridity index changes. On the other hand, the change in the evaporative ratio based on the direct CMIP6 outputs of actual evaporation Δ(E/P) is the vertical component of the movement of a hydrological basin due to the combination of all possible drivers of change. The difference between the estimates, Δ(E/P) − Δ(E*/P), determines if a hydrological basin follows or deviates from its original 1901–1950 Budyko curve.

### 2.2 Movements in the Budyko Space

Movement in Budyko space is the vector resulting from the change in the aridity index Δ(E_0_/P) and the evaporative ratio Δ(E/P) between the 50‐year means of two periods (Figure 1) (Destouni et al., 2013; Jaramillo & Destouni, 2014; van der Velde et al., 2014). The direction of movement corresponding to the reported CMIP6 output of actual evaporation is calculated as

(9) $$\theta =k-\arctan\left(\frac{\Delta (E/P)}{\Delta (E_{0}/P)}\right)$$

where θ is the direction in degrees, 0°<*θ*<360°, clockwise and from the upper vertical, *k* = 90° when Δ(E_0_/P) > 0 and k = 270° when Δ(E_0_/P) < 0. Conversely, the expected direction of movement along the Budyko curve expected only from aridity index changes (θ*) is

(10) $$\theta^{*}=k-\arctan\left(\frac{\Delta (E^{*}/P)}{\Delta (E_{0}/P)}\right)$$

In turn, the intensity of the movement (I) is calculated for both types of movement as

(11) $$I=\sqrt{{{(\Delta \left(E/P\right))}^{2}+\left(\Delta \left(E_{0}/P\right.)\right)}^{2}}$$

and

(12) $$I^{*}=\sqrt{{{\left(\Delta \left(E^{*}/P\right))\right.}^{2}+\left(\Delta \left(E_{0}/P\right.)\right)}^{2}},$$

respectively. Comparing the two types of movement can elucidate the drivers behind the observed long‐term Δ(E/P). For instance, if the CMIP6‐output movements (Equations 9 and 11) resemble those along the Budyko curve (Equations 10 and 12), we can ratify a significant role of long‐term changes in E_0_/P as a driver of changes in E/P. Otherwise, it can suggest significant contributions to Δ(E/P) from other drivers not represented in E_0_/P. However, it is worth noting that the influence of multiple drivers of change different from the aridity index may also counteract each other and result in a movement that follows the corresponding Budyko trajectory (Jaramillo & Destouni, 2014)

Finally, to quantify if a direction of movement follows its original Budyko curve (i.e., of 1901–1950), we assumed a deviation if the mean of the distributions of E/P between the two periods is statistically different from Δ(E*/P) (*p* < 0.05; unpaired *t*‐test). We used Jaramillo and Destouni's (2014) approach to illustrate movements in Budyko space as ‘windroses’ that summarize change for a large set of river basins; with green roses representing movements based on the CMIP6 output of E (Equations 9 and 11) and gray roses representing the movements expected from the trajectories described by each original Budyko curve (Equations 10 and 12).

## 3 Results

**Figure 2:**
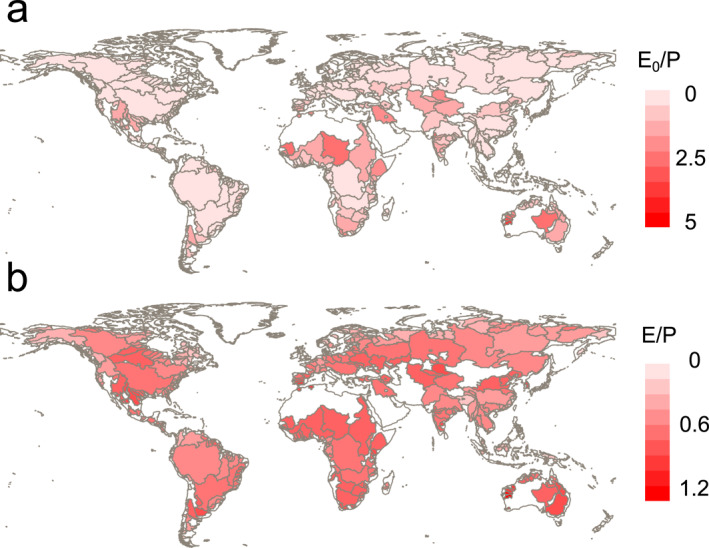
Original hydroclimatic conditions. The mean values from the seven models for a) aridity index (E_0‐EO_/P) and evaporative ratio (E/P) during the period 1901 to 1950 for 405 large river basins.

The selected 405 large river basins cover a wide variety of Earth's hydroclimatic conditions (Figure 2), from conditions where atmospheric energy demand is low and precipitation high─ such as those in Scandinavia or Canada─ to the opposite conditions such as the Sahel and Australia (Figure 2). In total, 258 river basins are energy‐limited or wet since E_0_/P < 1, and 147 are water‐limited or dry (E_0_/P >1). Generally, river basins with low E_0_/P share low E/P conditions, and vice versa, following the water and energy availability describing the shape of the Budyko curves (Figure 1).

**Figure 3:**
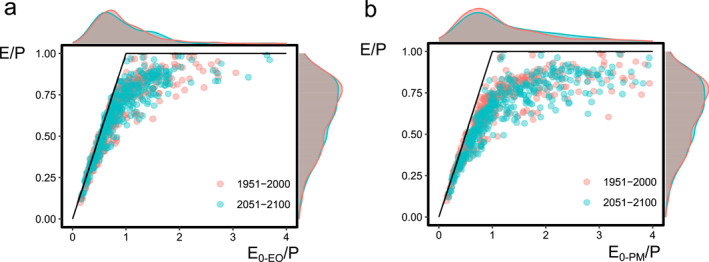
Location in Budyko space of the 405 river basins according to the 50‐year means in 1901–1950 and 2051–2100, with the water and energy limits constraining water and energy availability (black lines). Locations based on (a) E_0‐EO_/P and (b) E_0‐PM_/P. The few river basins with E_0_/P > 4 are excluded from the plot for better visualization.

Regardless of the estimate of potential evaporation used, most basins fall below the energy (E/P = E_0_/P) and water limits (E/P = 1), as expected from long‐term water and energy availability (Figures 3a and 3b). We find that the river basins are closer to the energy limit with the energy‐only estimate of potential evaporation (E_0‐EO_), resulting in lower aridity indexes. Moreover, the normal distributions of E/P and E_0_/P are skewed toward high E/P, where precipitation partitions more to E than runoff, and toward low E_0_/P, respectively. Regarding the change in location of Budyko space from 1901–1950 to 2051–2100, some river basins shift toward higher E_0_/P (Figures 3a and 3b), which is more evident with the E_0‐PM_ estimate. The higher temperatures of the future due to a decrease in the outgoing longwave radiation and an increase in absorbed solar radiation can explain this shift. Regarding changes in the distribution of E/P, a decrease in the peak is notable in the future (E/P ≈ 0.78).

**Figure 4:**
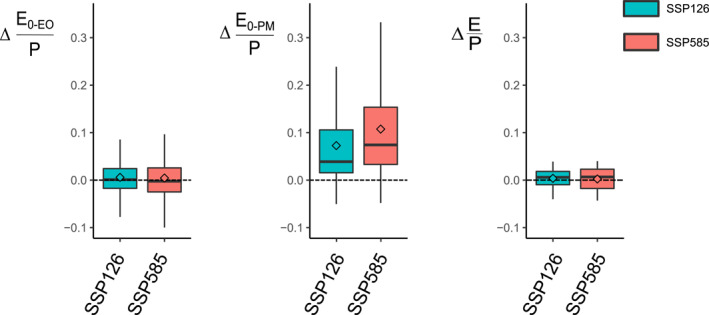
CMIP6 changes from 1901–1950 to 2051–2100 (dimensionless) in the aridity index based on the energy‐only, Δ(E_0‐EO_/P), and the Penman‐Monteith for open water, Δ(E_0‐PM_/P), and changes in the evaporative ratio Δ(E/P), for the SSP126 and SSP585 scenarios. Boxes represent the interquartile range (IQR; 25 and 75‐quantiles), whiskers extend to the 10‐ and 90‐quantiles and diamonds are the arithmetic average of all river basins (*n* = 405). Outliers are removed for visualization purposes.

Interestingly, the increase in E_0‐EO_/P in some basins as found in the Budyko plots is not significant (*p* > 0.05; *t*‐test) under the low‐emission SSP126 and high‐emission SPS585 scenarios, nor there is a substantial difference between both scenarios (Figure 4). However, E_0‐PM_/P increases in both scenarios, with more river basins experiencing an increase under SSP585 than SSP126. The E_0‐PM_/P increase under both scenarios also contrasts with the negligible changes of the mean in E/P (*p* > 0.05; *t*‐test) under both scenarios.

**Figure 5:**
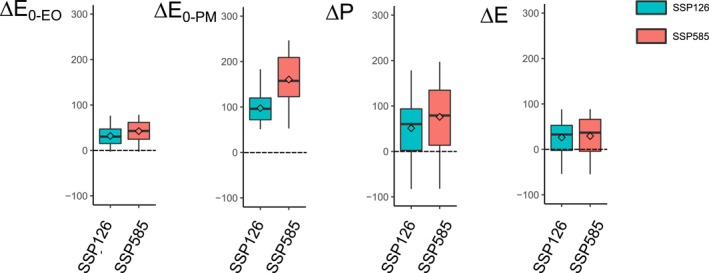
Hydroclimatic changes from 1901–1950 to 2051–2100 under the lowest SSP126 (blue) and highest SSP585 (red) CMIP6 emission scenarios. a) Changes in precipitation (ΔP; mm/yr), potential evaporation from the energy‐only (ΔE_0‐EO_; mm/yr), Penman‐Monteith for open water (ΔE_0‐PM_; mm/yr), and actual evaporation (ΔE; mm/yr).

The changes in the aridity index and evaporative ratio from 1901–1950 to 2051–2100 are related to the changes observed in P, E_0_, and E under global warming (Figure 5). For instance, most basins present small changes in E_0‐__EO_/P (Figure 4) as the increase in P from one period to the other is accompanied by a somehow large increase in P (Figure 5). For the case of the positive changes in E_0‐PM_/P, they rather arise from the increase in E_0‐PM_ being generally larger than the increase in P. Regardless of the greenhouse‐gas emission scenario, ΔE_0‐EO_ and ΔE_0‐PM_ are always positive, with ΔE_0‐PM_ changes being considerably larger than E_0‐EO_ changes (*p* < 0.05; *t*‐test). In addition, ΔP is positive for most river basins (75 percentile), and ΔE_0‐PM_ is higher in the SSP585 scenario than in the SSP126 scenario. However, there is no notable difference between both scenarios regarding ΔE from the CMIP6 outputs.

**Figure 6:**
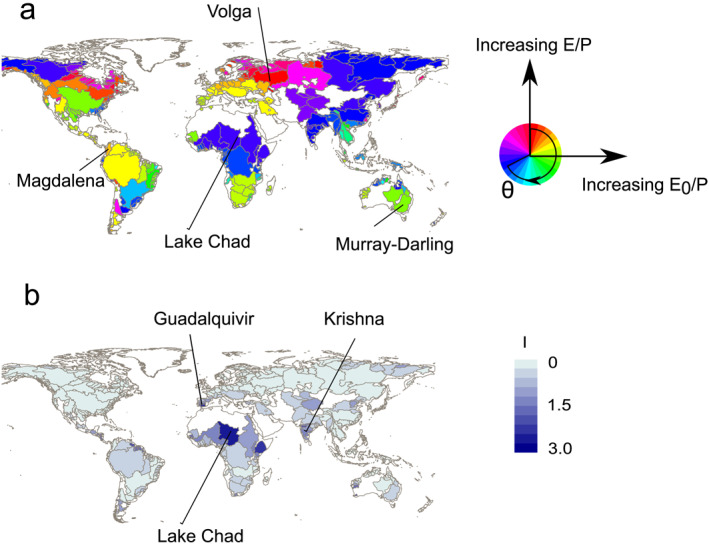
(a) The direction of movement (θ; Equation 9), and (b) intensity of movement in Budyko space (I; Equation 11) from 1901–1950–2051–2100, with the ssp585 scenario and energy‐only estimate of potential evaporation (E_0‐EO_).

The movements in the Budyko space of river basins worldwide cover the whole range of directions of movement and evidence continuous continental spatial patterns, especially across Eurasia (Figure 6). For example, from the Iberian peninsula, directions of movement are gradually changing eastwards from almost horizontal directions of movement with increasing E_0_/P (green)─ across upward movements in Eastern Europe (red and pink hues; e.g., Volga) ─ to horizontal movements with decreasing E_0_/P in Eastern Russia. However, this progressive spatial pattern of change of direction is not present in Africa, with Central Africa and the Sahel presenting opposite directions of simultaneous decreases in E_0_/P and E/P, and river basins in Southern Africa of increasing E_0_/P and E/P.

River basins such as the Murray Darling in Australia find an increase in E_0_/P accompanied by negligible changes in E/P, resulting in horizontal movements (Figure 6a; green). Conversely, the Lake Chad River basin moves in the opposite direction, as E_0_/P decreases and E/P presents negligible changes (Figure 6a; Lila). In another case study, the Magdalena River basin in Colombia presents a simultaneous increase in E_0_/P and E/P of similar magnitude, resulting in a diagonal change direction (*θ* = 45°). Regarding the intensity of movement, the largest gains are found in the tropical river basins in South America, basins of the Iberian peninsula and tropical and subtropical basins in Central Asia, as they move farthest in Budyko space (Figure 6b). Furthermore, the largest intensities of movement I occur with increasing E_0_/P and E/P (e.g., Guadalquivir); or decreasing E_0_/P and E/P (e.g., Lake Chad and Krishna basin).

Regarding movements along the Budyko curves, using both potential evaporation estimates yields good predictions of E/P for the period 1901–1950 when using the Budyko‐type (Yang et al., 2008) (Figure S2 in Supporting Information S1). The Lin's Concordance Correlation Coefficients (CCC; Lin et al. [2012]) are above 0.97 (i.e., given that a perfect match between E/P and E*/P is 1), explainable since most basins plot below the energy and water limits.

**Figure 7:**
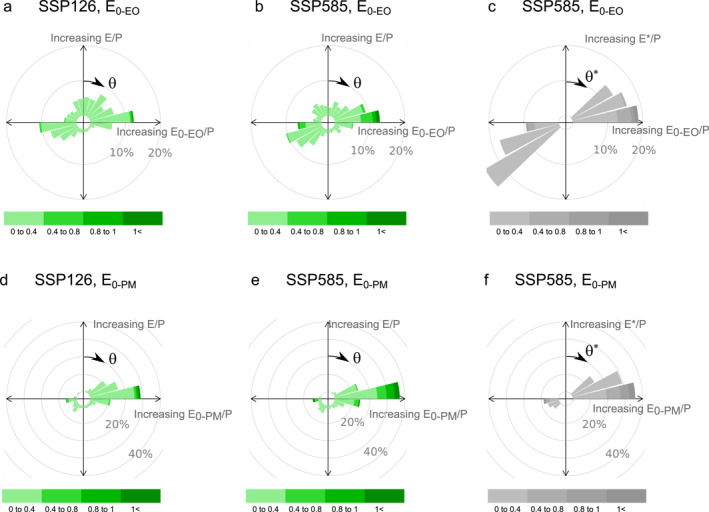
Roses of movement in Budyko space from 1901–1950 to 2051–2100 based on CMIP6 simulations (a, b, d, e; green roses) and according to the Budyko type model by Yang et al. (2008) (c, f; gray roses). Roses (a) and (e) are under the low‐emission RCP126 scenario and (b), (c), (e) and (f) under the high‐emission scenario RCP585. The range of directions of movement (0°<*θ*<360°) is divided into 15°‐interval paddles that group all basins moving in each direction interval, with directions (θ, θ*) starting from the upper vertical and clockwise. The color intervals represent the intensity of the movements (I, I*) in Budyko space in such a given direction.

The combination of θ and I resulting from hydroclimatic changes from 1901–1950 to 2051–2100 for the 405 basins are synthesized in roses (Figure 7), which are interpreted in the same way as typical wind roses of wind direction and speed. These roses summarize the combined effect of changes in E_0_/P and E/P. For the case of the lowest emissions scenario, SSP126, in which potential evaporation is expected to increase the least, river basins have moved across the entire spectrum of directions (Figure 7a). The 15‐degree range of directions in which most river basins moved was 75°<*θ*<90°, with 12% of all river basins (light and dark green; e.g., Murray‐Darling). Of these river basins, 1% moved with intensities larger than one (dark green). This range of direction of movement represents a combination of increasing E_0‐EO_/P with relatively smaller increases in E/P, resulting in almost horizontal movements in Budyko space. The rose for the corresponding high‐emissions scenario SSP585 looks similar but with higher intensities (Figure 7b).

Conversely, the corresponding movements derived from the Budyko‐type model and driven only by changes in the aridity index cover the ranges of directions 45°<θ*<90° and 225°<θ*<270° (Figure 7c), which are the possible range of slopes of movement of a river basin along the Budyko curve. By comparing both roses of movement (e.g., Figures 7b and 7c), it is evident that not all river basins are moving as expected from only changes in the aridity index. Movements in the directions 0°<θ*<45°, 90°<θ*<225° and 270°<θ*<360° can only occur by other drivers of change that are additional to aridity index changes. Regarding E_0‐EO_, 52% of river basins move in the ranges of directions 45°<θ*<90° and 225°<θ*<270° in SSP126 (Figures 7a) and 47% in SSP585 (Figure 7b).

The roses of the Penman‐Monteith estimate E_0‐PM_ evidence a larger number of basins moving horizontally to the right (75°<*θ*<90°), as E_0‐PM_/P increases much more than E/P (Figures 7d and 7e). For the SSP126 scenario, 28% of the basins move in this specific 15‐degree direction range (Figure 7d), while the number increases to 37% for SSP585 (Figure 7e). Furthermore, more basins appear to move in the range of directions 45°<θ*<90° and 225°<θ*<270°, 67% in SSP126 (Figures 7e) and 61% in SSP585 (Figure 7f). Hence, the difference in the range of directions of movement in Budyko space between greenhouse gas emissions scenarios, SSP126 and SSP585, is not as pronounced as between the two estimates of potential evaporation used to calculate them, E_0‐EO_/P and E_0‐PM_/P. It is worth noting that these results relate to the ensemble mean of the seven models; however, movements in Budyko space differ considerably across models, mostly due to their characteristics, settings, and structures (Figure S3 in Supporting Information S1). At least for the case of the ESM EC‐Earth3 and its dynamic vegetation version EC‐Earth3‐Veg, there appears to be no marked difference in the spectra of movement in Budyko space as their movement roses show negligible differences (Figures S3c and S3d in Supporting Information S1). This suggests that dynamic vegetation changes due to increasing greenhouse gas emissions do not affect their movement considerably in Budyko space as their direction and intensity remain similar.

**Figure 8:**
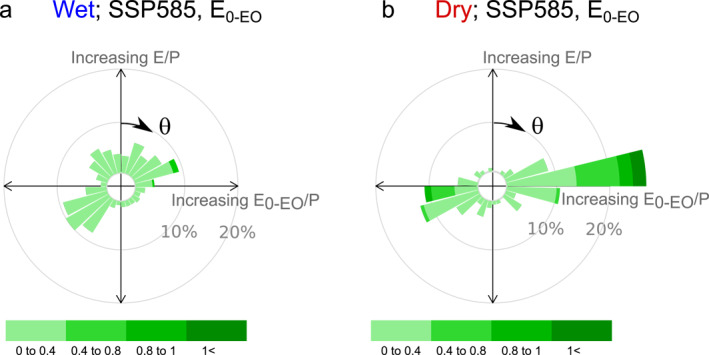
Roses for the groups of (a) wet or energy‐limited (E_0‐EO_/P<1) and (b) dry or water‐limited basins (E_0‐EO_/P>1). The roses correspond to the CMIP6 outputs, using the SSP585 scenario and with the energy‐only estimate of potential evaporation.

The movements in the Budyko space also depend on the initial conditions of aridity in each basin (Figure 8). For instance, wet basins tend to move more vertically than dry basins due to the asymptote existing in the water limit (E/P = 1; Figures 1 and 2). On the other hand, dry basins tend to move more horizontally (i.e., larger increases in E_0_/P than E/P) and have larger movement intensities as the aridity index is not constrained for higher aridity (Greve et al., 2015; Jaramillo & Destouni, 2014). Moreover, a larger percentage of dry basins finds an increase in E_0_/P compared to wet basins, for which there is an equal number of basins with increasing and decreasing E_0_/P.

Statistical analysis performed individually in each basin to determine if the river basin movements from 1901–1950 to 2051–2100 follow their original Budyko‐curve trajectories finds that regardless of the emission scenario and the potential evaporation estimate, most basins will not follow their Budyko curves (Table 2). This occurs as the difference between the 50‐year E/P means in 1901–1950 and 2051–2100 (i.e., ΔE/P) is statistically different than ΔE*/P in most basins (*p* < 0.05; unpaired t‐test). Moreover, regardless of the estimate of potential evaporation, the number of basins that follow their initial Budyko curves will decrease in SSP585 compared to SSP126. For the SSP126, at least 72% of the river basins will not follow their Budyko curves, increasing to 82% for the SSP585 scenario. Furthermore, the percentage of dry basins (ΔE_0_/P>1) not following their Budyko curves is usually lower than that of wet basins (ΔE_0_/P<1), indicating a tendency of energy‐limited basins to experience larger changes in E/P. Finally, more river basins will deviate from their Budyko trajectories when estimating E_0‐PM_ than E_0‐EO_.

**Table 2:** Nr. of Basins and Percentage Not Following Their Budyko‐Curve Trajectories From 1901–1950 to 2051–2100, Relative to 1901–1950 Conditions, According to the Mean CMIP6 Output, for Both SSP126 and SSP585 Scenarios, and Both Estimates of Potential Evaporation, E_0‐EO_ and E_0‐PM_

		Nr. Basins not following Budyko curve		% of the total in the group
SSP126	E_0‐EO_	Wet	191	74%
		Dry	99	67%
		Total	290	72%
	E_0‐PM_	Wet	218	84%
		Dry	102	69%
		Total	320	79%
SSP585	E_0‐EO_	Wet	211	81%
		Dry	121	82%
		Total	332	82%
	E_0‐PM_	Wet	238	92%
		Dry	117	80%
		Total	355	88%

*Note.* The percentages are calculated based on the number of wet (*n* = 258), dry (*n* = 147) and total (*n* = 405) number of river basins.

**Figure 9:**
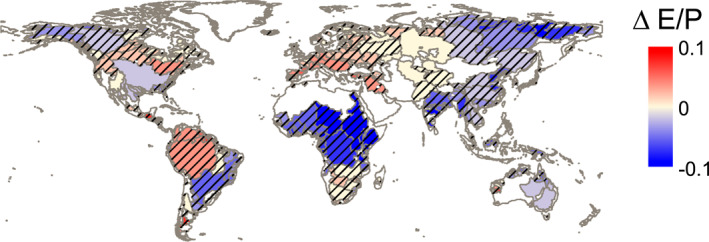
The river basin movements from 1901–1950 to 2051–2100 that do not follow their original Budyko curves (1901–1950) in SSP585 with both potential evaporation estimates are shown with a hatched pattern (*p* < 0.05, unpaired *t*‐test). Colors indicate the change in the evaporative ratio (ΔE/P) with the CMIP6 outputs of actual evaporation.

In total, 302 river basins (75%) will not follow their original Budyko curves under the SSP585 high‐emissions scenario regardless of the potential evaporation model used (Figure 9, not in Table 2). These basins are distributed across all continents and include river basins where E/P will decrease (blue) and increase (red). In Europe and Africa, all basins will not follow their Budyko curves. On the contrary, the few river basins that will still follow their Budyko curves include the Mississippi in the United States, the Murray‐Darling in Australia, and Ob in Asia.

## 4 Discussion

We find considerable differences of movement in Budyko space regarding direction and intensity when using the two estimates of potential evaporation; the energy‐only method (Milly & Dunne, 2016) and the Penman‐Monteith method for open water (Maidment, 1993). Estimates of potential evaporation by the latter type of models have been found to overpredict potential evaporation largely and their changes into the future over well‐watered surfaces, as they fail to capture changing stomatal conductance, resulting in biased estimates (Greve et al., 2019; Milly & Dunne, 2016; Yang & Roderick, 2019). This is noticeable from our results when using the Penman‐Monteith method for open water; changes in the aridity index appear to be unrelated to changes in the evaporative ratio (Figure 4). More recent modifications of these models have been proposed to account for these changes, such as the modified reference crop model of Yang et al. (2019). Nevertheless, there is a general deviation of river basins from their original Budyko‐type curves regardless of the potential evaporation estimate and across the seven CMIP6 models. The deviations occur because the estimates of the evaporative ratio from the CMIP6 outputs are statistically different (*p* < 0.05) from the estimates obtained by the Budyko‐type model selected (i.e., Equation 8; Yang, 2008).

Past global studies calculating movement in Budyko space based on precipitation, temperature, and runoff observations have found that 74% of almost 900 river basins worldwide deviated from their Budyko curves in the twentieth century (Jaramillo & Destouni, 2014). This study finds a similar percentage of basins deviating from their Budyko curves. However, the method used to estimate the deviations is not the same; Jaramillo and Destouni (2014) quantified deviations based on an analysis of roses of direction and intensity of movement in Budyko space (e.g., roses of Figure 7). This study goes beyond and determines for each basin when these deviations are statistically significant (Table 2 and Figure 9).

A large number of basins not following their Budyko‐type trajectories leave food for thought for the premise that space‐for‐time substitution does not apply to the Budyko framework. River basins do not generally follow their Budyko curves. The question is whether they do not follow them because of non‐stationary conditions (Milly et al., 2008) or because these movements are random since eco‐hydrological processes impacting changes in E/P do not follow changes in the aridity index (Padrón et al., 2017). To illustrate this, a recent study in the contiguous United States shows that the n‐parameter of the Budyko models can change without related changes in landscape and catchments characteristics (Reaver et al., 2022). However, an earlier study shows that movements in Budyko space in (some of) these basins follow spatial patterns of reservoir impoundment and agricultural expansion (D. Wang & Hejazi, 2011). Many other studies find that movement in Budyko space relates to specific signals of human developments such as reservoir impoundment (Jaramillo & Destouni, 2015; Levi et al., 2015; Sun et al., 2021), forestry (Jaramillo et al., 2018), environmental impacts on forests (Renner et al., 2013), to name a few. As such, the space‐for‐time substitution must be valid, at least in these cases of the scientific literature where movement in Budyko space relates to particular drivers and is based on observations.

Hence, it is possible that the large percentage of deviations observed here can be attributed to the land use and climatic changes of each of the socioeconomic pathway scenarios used for the future (SSP). Although each of the seven CMIP6 models, whose outputs we have used in the study, consists of different structures, modeling assumptions, and routines, they all share the parametrization of the SSP126 and SSP585 scenarios. Each scenario estimates various harmonized socioeconomic, demographic, technological, lifestyle, policy, and institutional drivers over the next century, with direct and indirect implications for water demand and availability (Riahi et al., 2017). Land‐use changes could be responsible for some of these deviations, as they induce changes in the evaporative ratio, pushing long‐term movements beyond the range of slopes given by a typical Budyko curve.

The SSP126 and SSP585 scenarios assume different developments of land‐use change and vegetation derived from different narratives of regulations, demand, productivity, environmental impacts, trade, and agricultural and forestry development (Popp et al., 2017). For instance, cultivated land can expand or contract by hundreds of millions of hectares over this century, depending on the scenario. According to a sustainable land transformation, the low‐emission scenario, SSP126, assumes little pressure on land resources due to low population projections, healthy diets with limited food waste, and high agricultural productivity. In this scenario, the deviations from the Budyko trajectories should relate to the evaporation‐related implications of the increase in forest area and decrease in pasture coverage (Sterling et al., 2013). Under this scenario and the energy‐only estimate of potential evaporation, 72% of basins will not follow their original Budyko curve (Table 2). This is a large number of basins, considering that the scenario assumes fewer land‐use changes than the other scenarios and uses the model that better predicts potential evaporation from CMIP6 outputs (Greve et al., 2019; Milly & Dunne, 2016).

Besides land‐use change, other drivers of change in the evaporative ratio may also deviate basins from their Budyko curves. For example, long‐term intra‐annual changes in energy and water availability related to seasonality may also account for deviations from the Budyko curves (X. Chen et al., 2013; Zanardo et al., 2012); the evaporative ratio may gradually change if precipitation patterns shift within the year, even with the same total annual precipitation. For instance, if precipitation shifts from months of high to low potential evaporation, the amount of precipitation partitioning into actual evaporation will decrease, decreasing the evaporative ratio (Xing et al., 2018). Similarly, a precipitation shift from snow to rain due to higher temperatures in winter and spring will decrease runoff (Berghuijs et al., 2014, 2017; D. Zhang et al., 2015), which under constant conditions of annual precipitation will increase the evaporative ratio, moving basins upward in Budyko space.

Our study builds on using the outputs of seven CMIP6 models for water and energy fluxes. Hence, our results are subject to the precision, accuracy, and potential biases inherent in these models. Nevertheless, the CMIP6 provide robust estimates of the global and regional energy and water fluxes as they are within the ranges of the reference datasets used for validation, judging by its ensemble mean (Li et al., 2021). Moreover, CMIP6 outputs of precipitation intensity have been relatively improved compared to their CMIP5 pairs, with reduced dry biases (Kim et al., 2020). However, biases have also been found in predicting water and energy fluxes from the CMIP6 models. For instance, the ensemble median of the CMIP6 evaporation model outputs evidence a warm summertime bias, mainly in the central United States, that results in a negative evaporation bias and a corresponding positive temperature bias via local land‐atmosphere coupling (Dong et al., 2022). In addition, the evaporation estimates simulated by land surface models appear to be the most sensitive parameter to model physics, followed by precipitation. Finally, although robust, the ensemble median of the CMIP6 can overestimate the water and energy fluxes over land, with the largest disagreements between models and the reference data sets occurring in South America and the Tibetan Plateau (Li et al., 2021).

Our study found that under the high‐emission scenario SSP585, less basins will follow their Budyko curves than SSP126 (Table 2). This is not surprising as this scenario's inherent land cover changes will modify vegetation and the Earth's surface more than the SSP126 (Meinshausen et al., 2020), with a considerable increase in cropland and a decrease in pastures and forests, which may affect surface evaporation rates (Riahi et al., 2017). However, this is not noticed by analyzing the movements of all 405 basins together in roses; basins may still not follow their Budyko curves even if moving in the same 15‐degree range of directions as expected from only aridity index changes (Figure 7c). The number of petals used to draw the roses of movement in Budyko space may then yield an incorrect estimate on whether basins are moving or not according to their Budyko curves.

Adding to the complexity of the effects of land‐use change on movement in Budyko space is the consequence of the increased concentration of atmospheric CO_2_, which on the one hand, may increase biomass production and plant water use (Zeng et al., 2016); and on the other hand allow plants to photosynthesize with less water‐use, which reduces stomatal conductance and transpiration (Ainsworth & Rogers, 2007; Betts et al., 2007; Medlyn et al., 2001). The overall effect of increased atmospheric CO_2_ on evaporation is debated and remains a challenge across CMIP6 models (Jaramillo et al., 2018; Zeng et al., 2016; Y. Zhang et al., 2016; Zhu et al., 2016). This is why some studies account for the changes in CO_2_ levels to estimate potential evaporation, like the formulation of Yang et al. (2019) based on the reference crop evaporation method (Allen, 1998). An improved understanding of vegetation responses to global warming is essential for ecohydrological adaptation and coping strategies under future climate change (Singh et al., 2020; Yang et al., 2019).

To date, the interpretation of movement in Budyko space has been used for a large set of applications, such as to determine: (a) how hydroclimatic change manifests in different biomes (van der Velde et al., 2014), (b) hydroclimatic change global effects of water use and water footprint estimations (Jaramillo & Destouni, 2015; Sun et al., 2021), (c) the influence of forest characteristics on water yield resilience to climate warming (Creed et al., 2014), (d) hydroclimatic change and implications for land water management (Piemontese et al., 2019), (e) the existence of shifts in hydroclimatology (Heidari et al., 2021) and drought (Maurer et al., 2021), and (f) the hydrological effects of vegetation change (Z. Chen et al., 2021). Our study elucidates the implications of using CMIP6 outputs to recognize drivers of changes in water fluxes and water partitioning (i.e., the evaporative ratio) and its effects on applications of the Budyko framework for separation and identification of drivers and responses. These results imply that basin changes worldwide are seldom purely climatically driven, and are overwhelmingly often caused by land use change, water use, ecohydrological dynamics, and other non‐climatic factors.

## 5 Conclusions

We quantified the movement in the Budyko space of 405 river basins from 1901 to 2100 based on the outputs of seven models from the Coupled Model Intercomparison Project ‐ Phase 6 (CMIP6). In addition, we used two different potential evaporation models and studied movements in Budyko space in low‐emission (SSP126) and high‐emissions scenarios (SSP585). Regardless of the potential evaporation estimate and the scenario used, from 1901–1950 to 2051–2100, most river basins (>72%) will not follow their Budyko curves of original basin conditions (1901–1950). Furthermore, the number of basins that do not follow their Budyko trajectories increases from the SSP126 to the SSP585 scenario and across both energy and water‐limited river basins. Finally, the Penman‐Monteith model for open water results in more basins deviating from their Budyko trajectories than the energy‐only method.

## Supporting information

Supporting Information S1

## Data Availability

All data will be available on the Bolin Centre Database (https://bolin.su.se/data/), Stockholm University, by searching the title of the article or author.
