# Supplementary material for: Fewer Basins Will Follow Their Budyko Curves Under Global Warming and Fossil‐Fueled Development
Source: Water Resour Res. 2022 Aug 22;58(8):e2021WR031825. doi: 10.1029/2021WR031825 (PMC9539592; doi:10.1029/2021WR031825)
Supplement: Supplementary file 1 — Supporting Information S1 [file WRCR-58-e2021WR031825-s001.pdf]

Supporting Information template

Please use this template when formatting and submitting your Supporting Information.

This template serves as both a “table of contents” for the supporting information for your article and as a summary of files.

Once you have completed this template, you should delete this instruction page.

Overview

Please note that all supporting information will be peer reviewed with your manuscript. For more information, please see the Supporting Information Guidelines in Author Resources

Using this Template

Type or paste the appropriate text (title, author list, and corresponding authors) into the template below.

Contents of this document:

- All Supporting text and figures should be included in this document.
- Movie files and audio files should be uploaded separately, following AGU naming conventions. File name and descriptions for these should be included in this template.
- All references should be included in the reference list of the main paper so that they can be indexed, linked, and counted as citations. The reference section does not count toward length limits.

How to fill out this document:

- Insert supporting information content into each appropriate section of the template.
- Figures should appear above each caption.
- To add additional captions, simply copy and paste each sample caption as needed.
- You will be prompted to upload these files on the Upload Files tab during the submission process, using file type “Supporting Information (SI)”
- Data (displayed in tables) should only be shared in the Supporting Information section for the sole purpose of peer review.

Water Resources Research

Supporting Information for

Fewer Basins will Follow their Budyko Curves under Global Warming and Fossil-fueled Development

Fernando Jaramillo[1,2*], Luigi Piemontese[3], Wouter R. Berghuijs[4], Lan Wang-Erlandsson[5], Peter Greve[6], Zhenqian Wang[7]

[1] Department of Physical Geography and Bolin Centre for Climate Research, Stockholm University, Stockholm, SE–106 91, Sweden
[2] Baltic Sea Centre, Stockholm University, Stockholm, SE-106 91, Sweden
[3] Department of Agricultural, Environmental, Food and Forestry Science and Technology University of Florence, Florence, Italy
[4] Department of Earth Sciences, Free University Amsterdam, Amsterdam, Netherlands
[5] Stockholm Resilience Centre, Stockholm University, Stockholm, Sweden
[6] International Institute for Applied Systems Analysis, Water Program, Laxenburg, Austria
[7] Key Laboratory of Western China's Environmental Systems (Ministry of Education), College of Earth and Environmental Sciences, Lanzhou University, Lanzhou, 730000, China
* Corresponding author: Fernando Jaramillo (fernando.jaramillo@natgeo.su.se)

Contents of this file

Figures S1 to S3

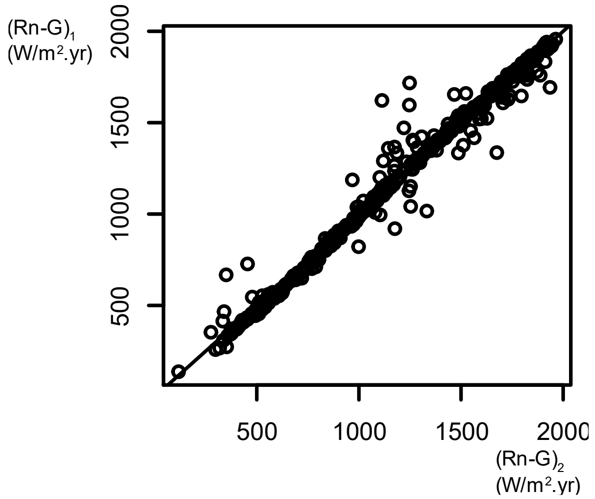

Figure S1. Figure S1. Coamprison of mean estimates of annual net radiation (Rn) minus ground heat flux (G), Rn-G for the period 1901-2100, as obtained from CMIP6 outpts of downwelling short (rsds) and long (rlds), and upwelling short (rsus) and long (rlus) wave radiation $(Rn\text{-}G)_1$ =rsds + rlds - rsus – rlus), and the sum of latent (hfls) and sensible (hfss) heat fluxes $(Rn\text{-}G)_2$ =hfls+hfss). For the mean of all seven CMIP6 models.

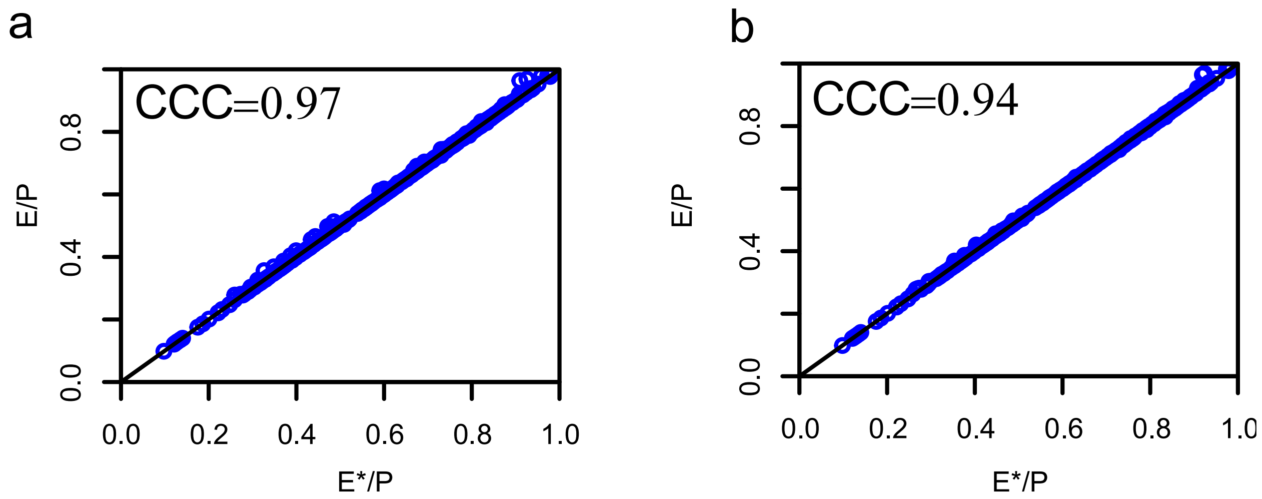

Figure S2. Comparison between the evaporative ratio obtained directly from actual evaporation outputs of the CMIP6 data (E/P) and the estimates from the Budyko-type model of Yang et al. (2008) (E^*/P; Eq. 8) for both a) $E_{0\text{-}EO}/P$ and b) $E_{0\text{-}PM}/P$.

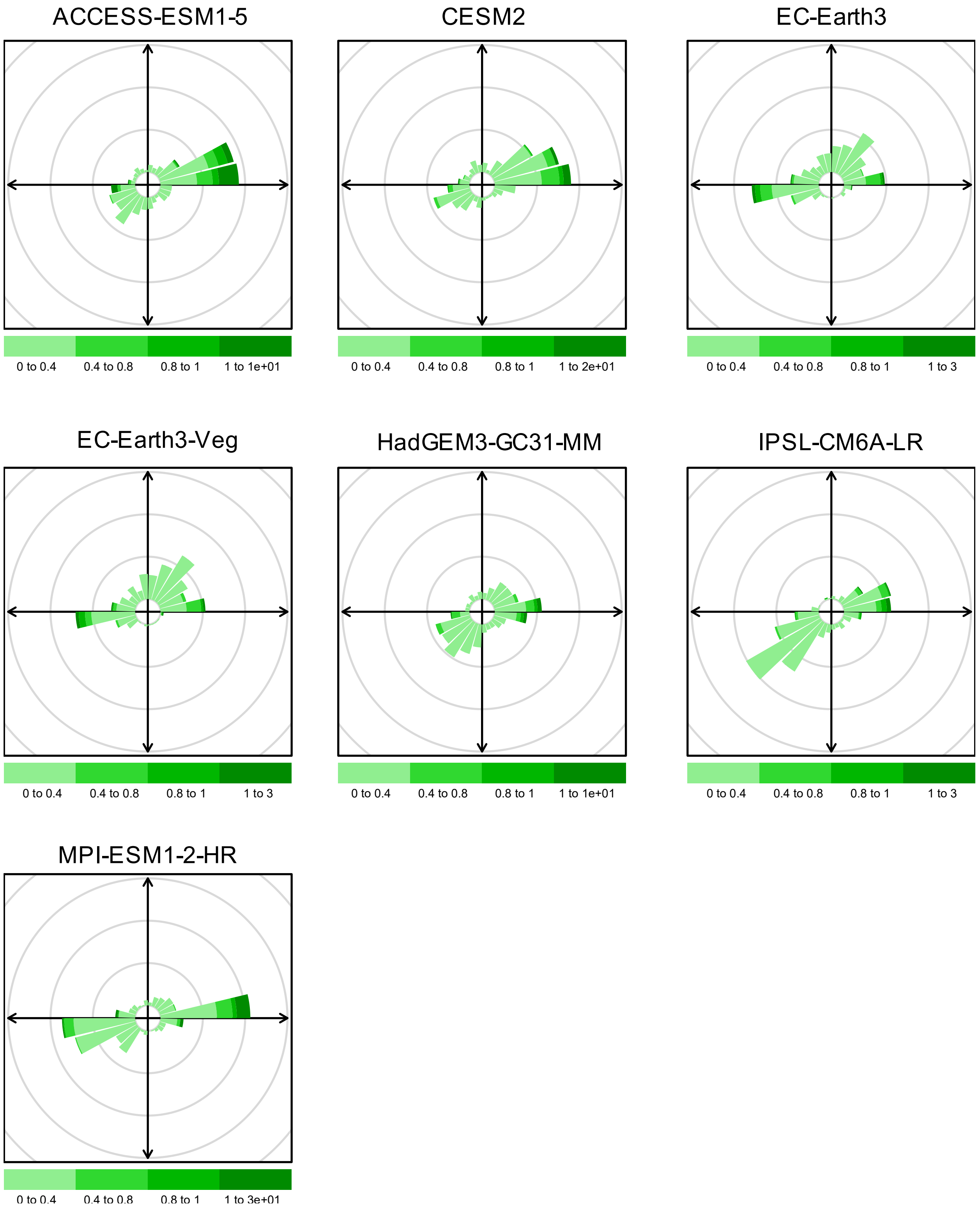

Figure S3. Roses of movement in Budyko space for the seven models used in the study. Greynlines visualize 10% intervals for the number of basins. See Fig. 7 for more details.
